# Crosstalk Between the Immune System and Plant-Derived Nanovesicles: A Study of Allergen Transporting

**DOI:** 10.3389/fbioe.2021.760730

**Published:** 2021-11-26

**Authors:** Christopher Stanly, Hyoseon Kim, Giuseppe Antonucci, Immacolata Fiume, Michele Guescini, Kwang Pyo Kim, Maria Antonietta Ciardiello, Ivana Giangrieco, Adriano Mari, Gabriella Pocsfalvi

**Affiliations:** ^1^ Institute of Biosciences and Bioresources, National Research Council of Italy, Naples, Italy; ^2^ Department of Applied Chemistry, Institute of Natural Science, Kyung Hee University, Yongin, South Korea; ^3^ Department of Biomedical Science and Technology, Kyung Hee Medical Science Research Institute, Kyung Hee University, Seoul, South Korea; ^4^ Department of Biomolecular Sciences, University of Urbino Carlo Bo, Urbino, Italy; ^5^ Allergy Data Laboratories (ADL), Latina, Italy; ^6^ Associated Centers for Molecular Allergology (CAAM), Rome, Italy

**Keywords:** strawberry, nanovesicles, allergens, proteomics, sequence similarity

## Abstract

**Background:** Nanometer-sized membrane-surrounded vesicles from different parts of plants including fruits are gaining increasing attention due to their anti-inflammatory and anticancer effects demonstrated by *in vitro* and *in vivo* studies, and as nanovectors for molecular delivery of exogenous substances. These nanomaterials are very complex and contain a diverse arsenal of bioactive molecules, such as nucleic acids, proteins, and lipids. Our knowledge about the transport of allergens in vesicles isolated from plant food is limited today.

**Methods:** Here, to investigate the allergenicity of strawberry-derived microvesicles (MVs), nanovesicles (NVs), and subpopulations of NV, we have set up a multidisciplinary approach. The strategy combines proteomics-based protein identification, immunological investigations, bioinformatics, and data mining to gain biological insights useful to evaluate the presence of potential allergens and the immunoglobulin E (IgE) inhibitory activity of vesicle preparations.

**Results:** Immunological test showed that several proteins of strawberry-derived vesicles compete for IgE binding with allergens spotted on the FABER biochip. This includes the known strawberry allergens Fra a 1, Fra a 3, and Fra a 4, and also other IgE-binding proteins not yet described as allergens in this food, such as gibberellin-regulated proteins, 2S albumin, pectate lyase, and trypsin inhibitors. Proteomics identified homologous sequences of the three strawberry allergens and their isoforms in total protein extract (TPE) but only Fra a 1 and Fra a 4 in the vesicle samples. Label-free quantitative proteomic analysis revealed no significant enrichment of these proteins in strawberry vesicles with respect to TPE.

**Conclusion:** Immunological tests and bioinformatics analysis of proteomics data sets revealed that MVs and NVs isolated from strawberries can carry functional allergens their isoforms as well as proteins potentially allergenic based on their structural features. This should be considered when these new nanomaterials are used for human nutraceutical or biomedical applications.

## Introduction

Extracellular vesicles (EVs) are nanosized lipid bilayer-surrounded vesicles that are released by cells. They are packed with a multimolecular cargo of proteins, lipids, nucleic acids, and small molecules, which makes them a valuable source of information on the cell of origin. EVs can be uptaken by local or distant cells with which they can interact. In this way, they contribute to cell–cell communication with important functional consequences. Membrane-bound vesicles that are similar to the mammalian EVs in size, shape, and density have been isolated and studied from various plant sources (Mu et al., 2014) (Zhang et al., 2011) (Perut et al., 2021) (Pocsfalvi et al., 2018) (Bokka et al., 2020). Recently, substantial attention has been given to plant-derived vesicles because of their frequently observed anti-inflammatory and anticancer activities in cellular and animal models (Mu et al., 2014) (Zhang et al., 2011) (Perut et al., 2021). Like EVs secreted by mammalian cells, vesicles in plants play an important role in intracellular and cell–cell communications, and according to recent hypothesis, they can take part in interspecies communication too (Mu et al., 2014). Recently, nanovesicles (NVs) were successfully isolated from strawberries using widely employed differential centrifugation (DC) and filtration-based methods (Perut et al., 2021). The NVs were shown to have the typical morphology and size distribution of mammalian EVs. They also found that the internalization of these vesicles by cells did not affect cell viability negatively and prevented oxidative stress in a dose-dependent manner.

**GRAPHICAL ABSTRACT:**
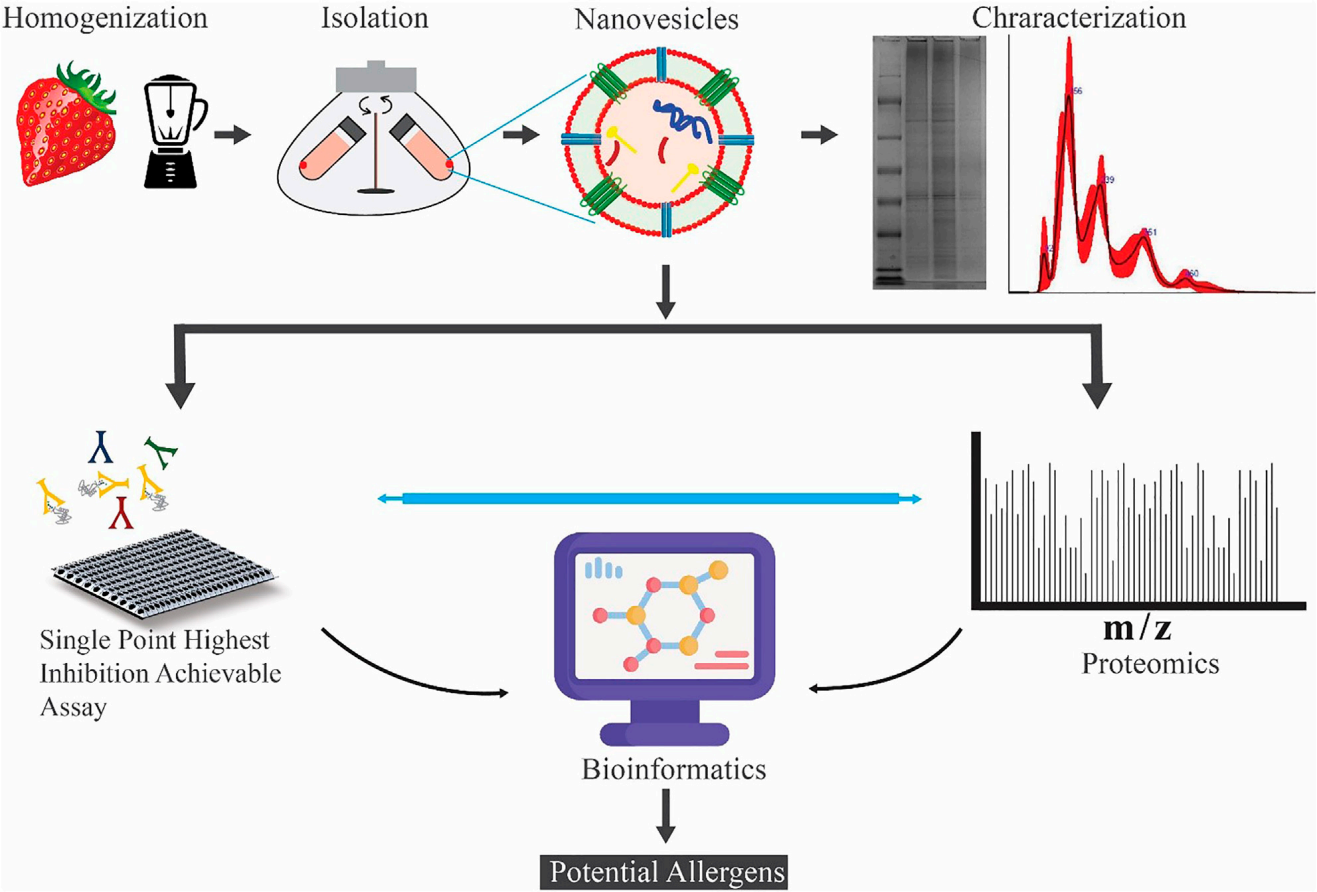


Strawberries are berries cultivated and consumed worldwide because they represent a delicious and nutritious food. Taxonomically, they belong to the genus *Fragaria*, included in the *Rosaceae* family. *Fragaria vesca*, known as wild strawberry, is a species with small berries, whereas *Fragaria ananassa* has higher size berries, and it is widely present on the market. Strawberries are described as false fruits bearing on the surface the real fruits, namely, the achenes, where the development of seeds occurs. The NCBI database reports 32,886 protein sequences of *F. vesca*, while only 1,671 records are present for the less sequenced and annotated *F. x ananassa* (dated July 26, 2021).

Strawberry extracts were shown to be rich in polyphenols, and they exhibit important biological activities *in vitro* against different cancer cell lines and *in vivo* against invasive breast cancer cells (Amatori et al., 2016) (Perut et al., 2021). Strawberries can also trigger allergic reactions in some people sensitized to this food (Hyun and Kim, 2011; Muñoz et al., 2010). There are three known strawberry allergens (*F. x ananassa*) that have been registered by the World Health Organization and International Union of Immunological Societies (WHO/IUIS, www.allergen.org) (Pomes et al., 2018) with the names Fra 1, Fra a 3, and Fra a 4 (Zuidmeer et al., 2006). Fra a 1 is a 17-kDa protein homologous to the major birch pollen allergen Bet v 1, a member of the ubiquitous PR-10 family of plant pathogenesis-related proteins. Fra a 3 is a 9-kDa lipid transfer protein (LTP), belonging to the PR-14 protein family. Fra a 4 is a 13-kDa protein belonging to the profilin family, the members of which play a key role in cell physiology. Profilins are highly cross-reacting allergens. This property is linked to the high conservation of their structural features highlighted by the high amino acid sequence identity between homologous molecules (Offermann et al., 2016). Profilins and Bet v 1-like proteins are classified as class 2 food allergens that are heat-labile, easily degraded by the gastrointestinal proteases, and responsible for localized oral allergy symptoms (OAS) (Alessandri et al., 2020). In contrast, LTP belongs to class 1 food allergens, which are heat and protease stable. They are clinically relevant allergens because their ingestion, inhalation, and skin contact can cause symptoms ranging from mild to very severe, including food-dependent exercise-induced anaphylaxis and anaphylactic shock. Fra a 1, Fra a 3, and Fra a 4 belong to multigene families, and therefore, their various isoallergens/isoforms are also reported in protein databases. In particular, 12 Fra a 1, 7 Fra a 3, and 2 Fra a 4 isoallergens/isoforms are reported today in UniProt protein database (July 26, 2021). Interestingly, the protein homologous of Fra 1, Fra a 3, and Fra a 4 have also been identified in *F. vesca* (Hyun and Kim, 2011), but they are not available in public protein databases.

In this study, we investigate if strawberry-derived vesicles carry protein sequences having homologs to known allergens using a quantitative proteomics and bioinformatics approach. Label-free shotgun proteomics was used to identify and quantify proteins in the total protein extract, and three vesicle isolates, microvesicles (MVs), NVs, and the density gradient ultracentrifugation (DGUC) separated NVs. BLAST searches against the COMPARE allergen and the UniProt *Fragaria* x *ananassa* databases were used to point out already known and potential allergens among the identified proteins based on structural features. Parallel to the proteomics analysis, we used the Single Point Highest Inhibition Achievable assay (SPHIAa) method associated with the FABER test (Tuppo et al., 2019) (Tuppo et al., 2018; Giangrieco et al., 2019) to identify the presence of functional allergens. FABER is a nanotechnology-based multiplex *in vitro* serological test, which takes advantage of database and bioinformatics tools of the Allergome platform (http://www.allergome.org/). FABER test exploits a comprehensive panel of 244 allergens, including the most important allergy markers, in addition to exclusive allergens not available in other test systems. Finally, the results obtained from FABER and the quantitative proteomics were merged to evaluate the enrichment of the potential allergens in the different vesicle fractions with respect to the total proteins extracted from strawberries.

## Materials and Methods

### Fruit Material and Isolation of Nanovesicles by Differential Ultracentrifugation

Strawberries grown without any pre- and postharvest treatments were acquired from a local vendor in Naples, Italy. 250 g of fresh fruits were weighed and washed thrice with cold running tap water and once with Milli-Q water. The calyx was removed, berries were air dried and transferred to a blender containing 1:1 weight-to-volume ratio extraction buffer composed of 100 mM phosphate and 10 mM ethylenediamine tetraacetic acid (EDTA) (pH 8), and protease inhibitor cocktail [0.25 ml leupeptin (1 mg/ ml), 1.25 ml 100 mM phenylmethylsulfonyl fluoride (PMSF), and 0.8 ml 1 M sodium azide for every 250 g of strawberry]. The sample was homogenized three times at a maximum velocity for 10 s. Crude vesicles were isolated by differential centrifugation (DC) following the procedure described by Stanly et al. (2016)*.* Briefly, sequential low-velocity centrifugations were performed at 400, 800, and 2,000 ×*g* using a swinging-out bucket rotor for 30 min for each step at 22°C. The supernatant was centrifuged at 15,000 ×*g* in a fixed-angle rotor for 30 min at 22°C to collect the pellet containing the microvesicles (MVs). The supernatant was centrifuged at 150,000 ×*g* for 60 min at 4°C using a Type 70 Ti Beckman rotor in a Beckman Coulter Optima L-90K ultracentrifuge. The pellet was resuspended in 28.5 ml of 50 mM Tris-HCl (pH 8.6) buffer and centrifuged at 150,000 ×*g* for 60 min at 4°C. The resulting Pellet (crude NVs) crude NVs were suspended in 50 mM Tris-HCl pH 8.6 and further separated by DGUC.

### Density Gradient Ultracentrifugation Using Iodixanol

DGUC was performed according to Karimi et al. (2018) with some modifications. OptiPrep^TM^ (60 w/v% iodixanol in distilled water; Axis-Shield, Oslo, Norway) was diluted to 50%, 30%, and 10%, (w/v), and 3 ml of each solution was layered in 13.5-ml polypropylene centrifugation tubes (Beckman Coulter, Pasadena CA, United States). Three milliliters of the NVs isolated by dUC was gently layered on the top and centrifuged at 110,000 ×*g* for 24 h at 4°C using an SW 41 Ti Beckman rotor in a Beckman Coulter Optima L-90K ultracentrifuge. Twelve vesicle fractions (layers) were collected, and the density of each fraction was determined. The standard curve was prepared using 2 μL of iodixanol solution with concentrations between 1% and 50%. Standards and DGUC fraction samples previously diluted 5,000-fold in distilled water were measured using Nanodrop 2000 (Thermo Fisher Scientific Inc., Waltham, MA, United States) at 244 nm wavelength according to the instruction of the manufacturer. The density of samples was calculated based on their iodixanol concentration.

### Density Gradient Ultracentrifugation Using Sucrose/Deuterium Oxide Cushions

The sample was underlaid with a sucrose/D_2_O double cushion consisting of 6 ml of 1 M and 4 ml of 2 M sucrose prepared in 20 mM Tris pH 8.6/D_2_O in 38.5-ml thin-wall polypropylene tubes (Beckman Coulter, United States) and centrifuged at 110,000 ×*g* for 3 h at 4°C using an SW 28 Ti rotor (Beckman Coulter, United States). The fractions were collected by piercing the bottom of the tube, diluted in solubilization buffer, and centrifuged at 110,000 ×*g* for 1 h at 4°C using an SW 28 Ti rotor to remove sucrose. The resulting pellets were resuspended in 10–20 µl of solubilization buffers. Protein concentration was determined by the microbicinchoninic acid protein assay (Pierce/Thermo Scientific, Rockford, IL, United States) using a nanodrop 200 spectrophotometer (Thermo Fisher Scientific, Wilmington, DE, United States). Samples were stored at −80°C until used.

### Nanoparticle Tracking Analysis

Particle number concentration was measured by nanoparticle tracking analysis (NTA) using a Nanosight NS300 (Malvern Panalytical, United States) instrument. The samples were diluted to obtain less than 100 particles per frame; then 5 × 60-s measurements were performed with a moderate flow and analyzed by the built-in software of the instrument (NanoSight NTA software 3.4 version 003).

### Preparation of Total Protein Extract of Strawberries

Strawberries were frozen using liquid N_2_ and then transferred into a stainless-steel blender. The sample was homogenized using the blender at maximum velocity for 10 s three times. In between blending intervals, some liquid N_2_ was added to grind the fruit completely into a powder. Two grams of powdered fruit was weighed and transferred into a 50-ml test tube. Six milliliters of extraction buffer [2% SDS, 60 mM dithiothreitol (DTT), 20% glycerol, and 40 mM Tris-HCl, pH 8.5] was added per gram of powdered fruit and incubated at 90°C for 8 min. After incubation, the sample was centrifuged at 8,000 × *g* for 15 min at 4°C using a fixed angle rotor. The supernatant was collected in a 50-ml Falcon tube, and 25 ml of precipitation solution [(containing 10% w/v) trichloroacetic acid and 20 mM DTT prepared in ice-cold acetone) was added. The solution was kept at −20°C for 45 min and then centrifuged at 18,000 ×*g* for 10 min at 4°C. The supernatant was discarded, and the pellet was washed three times with a 7.5-ml washing solution containing 20 mM DTT prepared in ice-cold acetone. The pellet was suspended in 35 ml of washing solution, incubated at -20°C for 1 h, and centrifuged at 20,000 x*g* for 10 min at 4°C. The supernatant was discarded, and the pellet was air dried. The air-dried pellet was resuspended in 700 μl of rehydration buffer containing 7 M urea, 2 M thiourea, 30 mM Tris HCl, pH 8.5, and 43 mM DTT. The sample was kept at constant shaking for 1 h at room temperature and centrifuged at 12,000 × *g* for 10 min at room temperature. The resulting supernatant was used as the total protein extract (TPE).

### Protein Quantification and Profiling

The protein concentrations were measured by a microbicinchoninic (BCA) acid assay kit (Thermo Scientific, Rockford, IL, United States) using a Nanodrop 2000 spectrophotometer (Thermo Fisher Scientific Inc., Waltham, MA, United States). Sodium dodecyl sulfate-polyacrylamide gel electrophoresis (SDS-PAGE) was used for protein separation. Samples (20 μg protein measured by the micro-BCA assay) were electrophoretically separated under reducing conditions on a precast Novex Bolt 4–12% Bis-Tris Plus gel using Bolt MOPS SDS running buffer (Life Technologies, Carlsbad, CA, United States) according to the instructions of the manufacturer and stained with colloidal Coomassie blue (Applichem GmbH, Darmstadt, Germany).

### Lysis of the Vesicles and Enzymatic Digestion

For in-solution digestion proteomics analysis, samples (50 μg of protein measured by micro-BCA assay) were resuspended in 0.2% RapiGest SF (Waters Corp., Milford, MA, United States), and vesicles were lysed using five freeze and thaw cycles under sonication. Proteins were reduced using 5 mM DTT (Sigma-Aldrich, Saint Louis, MO, United States), alkylated using 15 mM iodoacetamide (Sigma-Aldrich, Saint Louis, MO, United States), and proteolytically digested using mass spectrometry-grade trypsin (Pierce, Thermo Fisher Sci Rockford, IL, United States). Samples were vacuum dried and solubilized in 5% acetonitrile and 0.5% formic acid (LC-MS grade, Thermo Sci. Rockford, IL, United States) before nano-HPLC-MS/MS analysis.

### LC-ESI-MS/MS

The peptide samples were dissolved in 0.1% formic acid by gentle vortexing. Shotgun proteomics analysis was carried out using an Acquity UPLC system (Waters, Milford, MA, United States) directly coupled to a Q-Exactive Orbitrap Hybrid Mass Spectrometer (Thermo Fisher Scientific). For the proteome profiling analysis, the optimized gradient elution program of solvent A (Sol A: 0.1% formic acid) and solvent B (Sol B: 0.1% formic acid in acetonitrile) was set as follows: (T min/% of Sol B): 0/5, 4/5, 9/12, 50/30, 110/40, 112/80, 118/80, 120/5, 140/5. The dissolved peptides were separated using an EASY-spray column (50 cm × 75 µm i.d., C18, 2 μm, 100 Å, Thermo Fisher Scientific). MS acquisitions were performed in positive ion mode using an electrospray voltage of 2.4 kV. The operating conditions for full MS scan were set as follows: scan range, 400–2,000 m*/z*; resolution, 70,000; automated gain control target value (AGC target), 3.0 × 10^6^; maximum ion injection time (IT), 100 ms. The operating conditions for MS/MS scan were set as follows: resolution, 17,500; AGC target, 5.0 × 10^5^; minimum AGC target, 2.0 × 10^3^; maximum IT, 50 ms; normalized HCD collision energy (NCE), 30; dynamic exclusion time, 30 s; isolation window, 2.0 *m/z*. Samples were analyzed in three technical replicates.

### Protein Identification and Quantitation

The acquired raw LC-ESI-MS/MS data were processed using the SEQUEST HT search engine of the Proteome Discoverer v.2.4 program (Thermo Fisher Scientific). The tandem MS spectra were analyzed against the *Fragaria vesca* protein database (January 2020, 32,886 entries) in the NCBI database, which generated decoy spectra. The following SEQUEST search parameters were used: cleavage of arginine and lysine residues using trypsin, allows for two missed cleavages, carbamidomethylation of cysteines, variable modifications of oxidation of methionine and protein N-terminal acetylation. The threshold for identification of the target peptide spectrum was applied to a 1% false discovery rate (FDR) in protein level analysis. Tolerances were set at 30 ppm for precursor mass and 0.02 Da for fragment mass, respectively.

### IgE Inhibition Experiments with the FABER^®^ Testing System

FABER is a multiplex *in vitro* serological test allowing the detection of IgE antibodies specifically recognizing allergens immobilized on a biochip (Tuppo et al., 2018; Giangrieco et al., 2019). The FABER version used to perform this study (FABER 244–122–122) contains 122 purified allergens and 122 multiple protein allergenic extracts, coupled to chemically activated nanoparticles and then spotted on a biochip (Figure 3). The FABER biochip is used for an ELISA-like testing procedure that allows the detection of specific IgE to each of the 244 allergenic preparations. The final signal for each allergen is obtained with the use of an optical scanner and elaborated with specific software. The signals detected for each spot are interpolated with values obtained with an internal IgE standard reference curve present in each biochip and also used as a reference between chips. Signal intensities are then used to calculate the arbitrary units, FIU (FABER International Units), correlated with the level of IgE bound to each allergen spot. To obtain information on the content of possible allergens in the strawberry samples, the SPHIAa (Pasquariello et al., 2012; Tuppo et al., 2019) (Yakhlef et al., 2021) was performed on the FABER platform. Sera of allergic patients for which the IgE profile was already known was used as providers of IgE recognizing specific allergenic proteins spotted on the FABER biochip. The assay was performed by incubating 0.12 ml of the sera pool with 0.12 ml of a solution containing 0.1 mg of the NVs samples. The IgE-binding inhibition was evaluated by running the FABER test and recording the residual IgE binding on the allergens spotted on the biochip. Experiments were carried out in duplicates, and the mean values are reported. Reference values for lack of IgE-binding inhibition were obtained by running control samples where the allergen solution was substituted with buffer only. The inhibition values were calculated in real time by a specific procedure developed within the InterAll software (version 5.0, ADL s.r.l.) (Alessandri et al., 2017). Values equal to or higher than 30% inhibition were considered as reliable inhibitions and reported, whereas lower values were discarded.

#### Sera of Patients

Sera of patients that had proven to be sensitized to different plant foods and had been analyzed with the FABER test were selected. To carry out the SPHIAa assays (Figure 3), four sera of patients sensitized to different plant foods and containing IgE-recognizing relevant plant allergens, such as LTP, profilin, Bet v 1-like, GRP, and seed storage proteins, were selected in the InterAll databank (version 5.0, ADL S.r.l.) (Alessandri et al., 2017). A volume of 0.5 ml was collected from each one of the four sera and pooled to be used for IgE-binding inhibition experiments.

All patients gave their informed consent to the use of their clinical data for research purposes in an anonymous form. Because of the purely comparative nature of this study, along with the fact that all venous blood samplings were part of routine clinical practice and that a residual part of the routine sample was used for inhibition experiments, formal approval by the Ethical Committee was not necessary.

### Bioinformatics

The OmicsBox 2.0.10 software package was used for *in silico* data analysis (Conesa and Gotz, 2008). Protein sequences were blasted against i) the NCBI public database with taxonomy filter green plants (*Viridiplantae*, 20,952,768 entries) and ii) the COMPARE allergen database (2348 entries) (Van Ree et al., 2021) (https://comparedatabase.org/) using BlastP service with a maximum number of blast hits 20 and expectation value of 1.0 × 10^−3^. Blast databases of *F. x ananassa*, NCBI green plants, and COMPARE were created by OmicsBox 2.0.10. The InterPro domain searches were performed using the public European Molecular Biology Lab-European Bioinformatics Institute to identify sequences against the signatures of CDD, HAMAP, HMMpfam, HMMPIR, Fprintscan, and BlastproDom Interpro. All sequences generated InterPro results. Annotated sequences were mapped against exclusively created GO-annotated proteins to obtain functional labels of GO associated and ID mapping of Uniprot. The FunRich program was used to prepare Venn diagrams (Pathan et al., 2015). Sequence homologs of the three known strawberry allergens (Fra a 1, Fra a 3, and Fra a 4) were identified by blasting the sequences of *F. vesca* (wild strawberry, Taxonomy ID: 57,918) in the UniProt database used for the protein identification against *F. x ananassa* (Taxonomy ID: 3747, 1671 entries on July 21, 2021).

## Results

### Isolation and Characterization of Microvesicles and Nanovesicles

**FIGURE 1:**
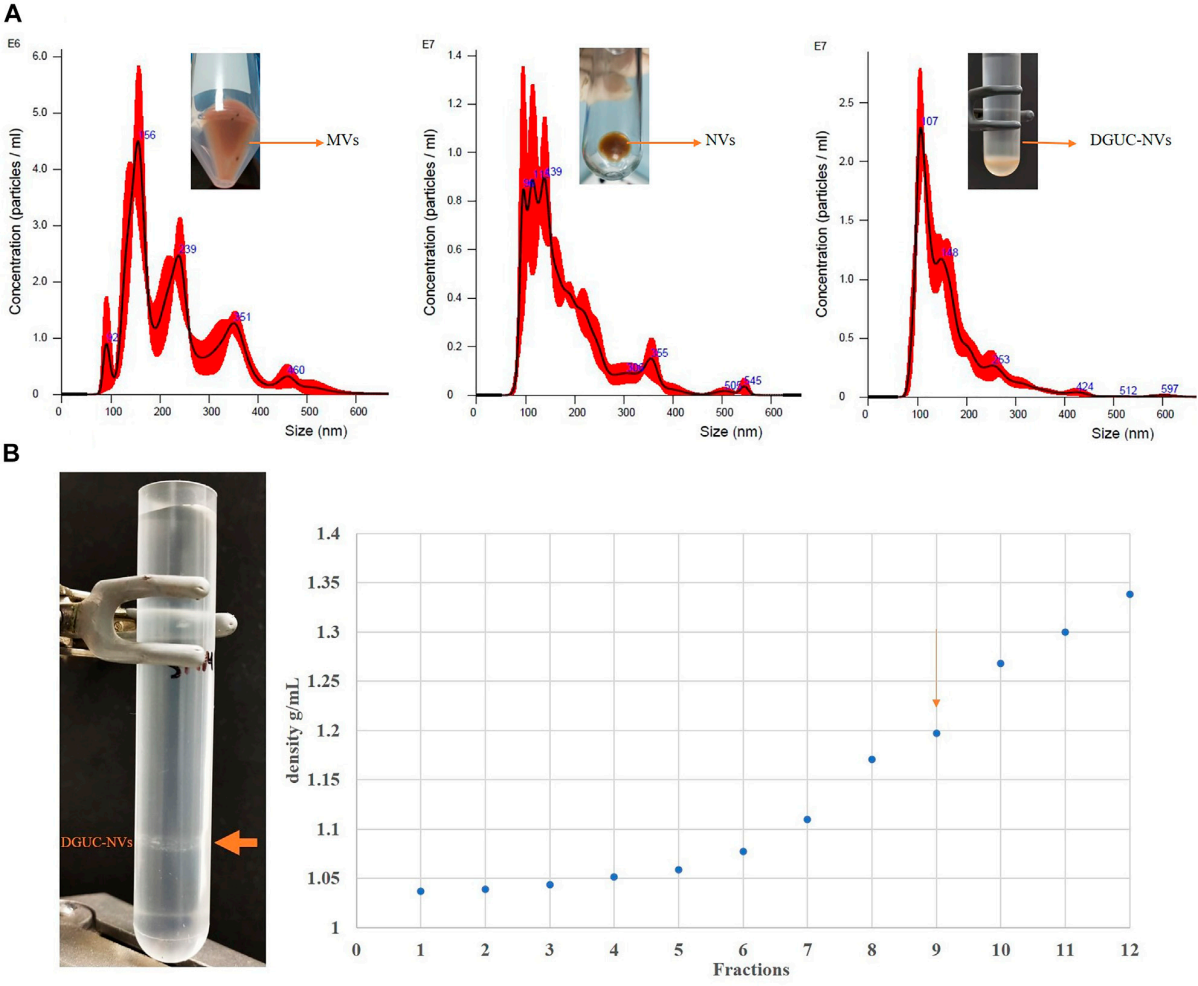
**(A)** Nanoparticle tracking analysis showing the size distribution of microvesicles (MVs), nanovesicles (NVs), and the sucrose density gradient purified nanovesicle fraction (DGUC-NVs). Particle numbers, mean diameter values, and standard deviations are reported in Table 1. **(B)** The pictures on the left show the sample of strawberry-derived NVs after iodixanol density gradient ultracentrifugation (DGUC). After DGUC, a single visible band could be observed (orange arrow). The densities measured in the 12 different fractions from top to bottom are shown in the chart on the right. The visible band was observed in fraction 9. The vesicles floating in fraction nine are similar in density to mammalian extracellular vesicles, which have a density of 1.13–1.19 g/ ml (Konoshenko et al., 2018).

MVs and crude NV samples from strawberries were isolated by DUC as low- (15,000 × *g*) and high-velocity (110,000 ×*g*) pellets according to the protocol (Supplementary Figure S1). NV samples were further separated by DGUC using iodixanol, a nonionic density gradient medium with relatively low osmolality and high density (Figure 1B), or sucrose/D_2_O cushions. The isolated vesicles were characterized regarding their size, density, zeta-potential, protein concentration, and bio-cargo composition according to the recent MISEV guidelines (Théry et al., 2018).

Figure 1A and Table 1 show the size distribution of particles in MVs, NVs, and the sucrose DGUC separated NV fraction (DGUC-NVs) analyzed by NTA. The size distribution of vesicles in the density gradient purified fraction (DGUC-NVs) was more homogenous with abundant small-sized (<150 nm) vesicles compared with MVs, which had a larger size distribution. The small vesicles that are present in the MV fraction could have cosedimented during the 15,000 ×*g* centrifugation step. The measured z-potential showed a negative surface charge of −26 mV for DGUC-NVs. The protein amounts associated with the vesicles were determined by the micro-BCA assay. The isolation (Supplementary Figure S1) yielded 20 mg of proteins in the MVs, 38 mg in the NVs, and 5 mg in the DGUC-NVs from 250 g of strawberry. The calculated particle per µg of protein ratio for the NVs, MV samples, and DGUC-NVs fraction were 8.0E8, 2.2E9, and 3.0E9 particles/µg of protein, respectively. The increased particles to protein ratios indicate the enrichment of the vesicles in the samples.

**TABLE 1:** The particle concentration and the size range of different vesicle populations isolated from strawberries.

Sample	Mean and standard deviation (nm)	Mean diameter of the most abundant particles (nm)	Major sub-populations (nm)	Minor sub-populations (nm)	Particle numbers/mL of the original juice
Microvesicles (MVs)	230.7 ± 4.7	156	92, 239, 351	460	1.9 * 10^12^
Nanovesicles (NVs)	179.3 ± 4.2	139	96, 115, 139	306, 355, 505, 545	8.2 * 10^12^
Sucrose density gradient purified nanovesicle fraction (DGUC-NVs)	165.3 ± 2.5	107	148	253, 424, 597	1.69 * 10^12^

### Proteomic Analysis of Vesicles Isolated from Strawberry

**FIGURE 2:**
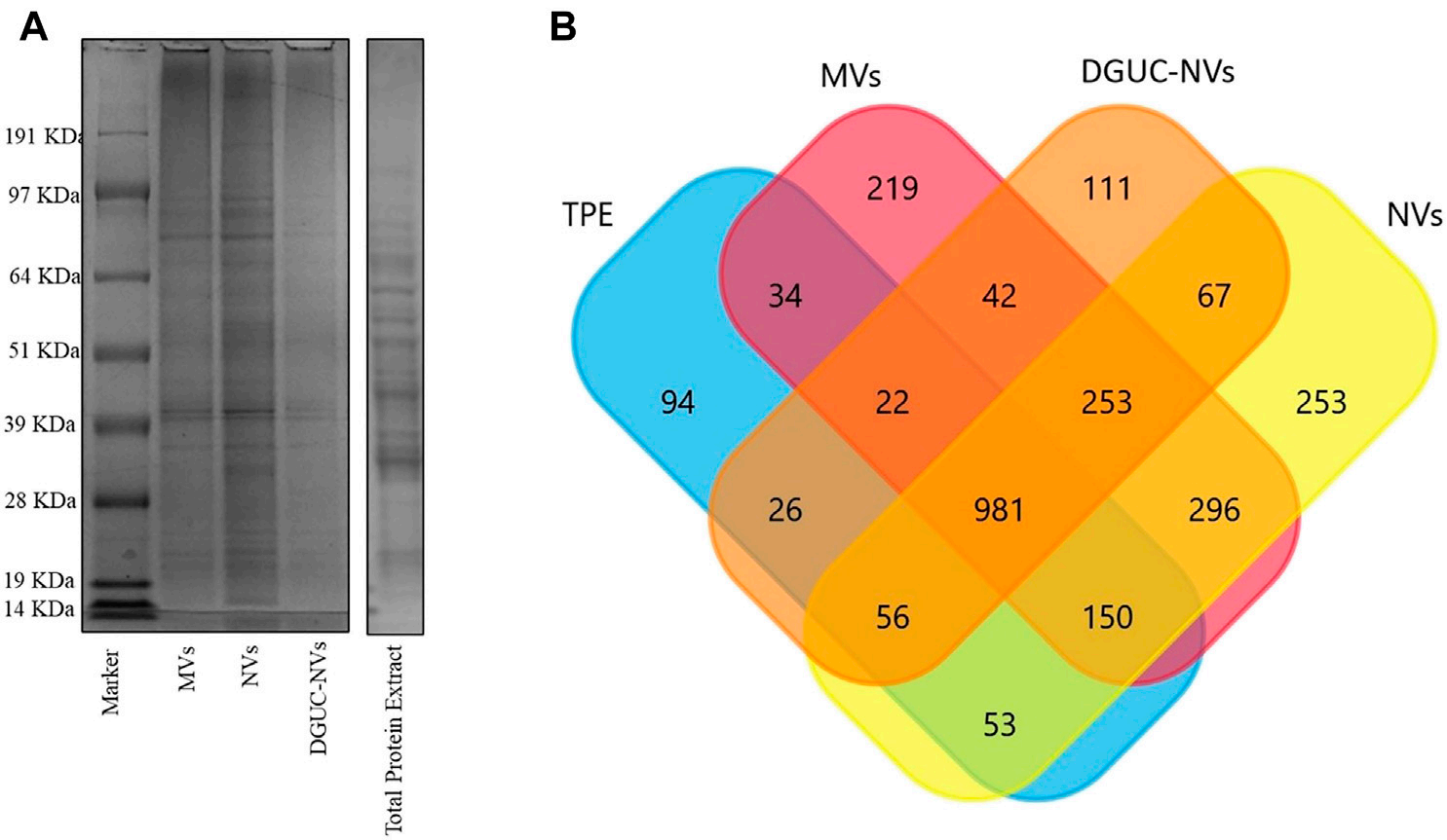
**(A)** SDS-PAGE protein profile of MVs, NVs, DGUC-NVs, and total protein extract (TPE). **(B)** Venn diagram showing the number of identified proteins and their overlappings in the TPE, MVs, crude NVs, and the sucrose gradient density DGUC-NV fraction.

Figure 2A shows the SDS-PAGE protein profile of the MVs, NVs, and DGUC-NVs samples isolated from strawberries. The gel image shows protein profiles with numerous bands that are evenly distributed throughout the gel. The three vesicle samples exhibit similar SDS-PAGE protein profiles different from that of TPE.

Samples TPE, MVs, NVs, and DGUC-NVs were subjected to in-solution digestion-based label-free shotgun proteomic analysis. Supplementary Table S1 reports the proteins identified against the *F. vesca* NCBI database in each sample, and Figure 2B shows the numbers of identified proteins. Proteins (981) were commonly identified in all samples, while 219, 253, and 111 sequences were unique to MVs, NVs, and DGUC-NVs, respectively (Supplementary Table S1). Vesicle isolates express highly abundant heat shock proteins (HSP83, HSP70, and HSP90), villin-3-like protein, myosin-10, ATPases, and clathrin heavy chain-1 protein. However, it should be noted that these proteins were also abundant in the TPE. A label-free quantitative study was performed to highlight the differentially expressed proteins over the three vesicle isolates (MVs, NVs, and DGUC-NVs) with respect to TPE. Four hundred one commonly expressed proteins were quantified over the sample set (Supplementary Table S2). Volcano plots highlight the statistically significant differential expression according to the *t*-test (Supplementary Table S2). There are 75 MVs, 30 NVs, and 51 DGUC-NVs proteins that showed significantly higher expression levels compared with their expression in TPE (Supplementary Table S3). In particular, six proteins were enriched more than two times in all the vesicle samples (MVs, NVs, and DGUC-NVs), and these were tubulin beta chain, protein SSUH2 homolog, HSP90, pyruvate kinase (PK), malonyl-CoA decarboxylase, and a probable mediator of RNA polymerase II transcription. The function of SSUH2 homolog proteins in the plant is largely unknown, and its increased expression in vesicles would need further investigation. HSP90 and the glycolytic enzymes PK are frequently identified also in mammalian cell-derived EVs. Malonyl-CoA decarboxylase is involved in fatty acid biosynthesis and, its expression, secretion, or transportation in vesicles has not been reported, although it is known that some EVs carry proteins involved in lipid metabolism including different enzymes (Clement et al., 2020). ATPases, similar to other plant-derived vesicles, such as, for example, that from citrus juice fruit (Pocsfalvi et al., 2018) and tomato fruit (Bokka et al., 2020), were also significantly enriched in strawberry vesicles (Supplementary Table S3). The specificity of these six proteins enriched in strawberry vesicles could be further elucidated as general markers for fruit-derived vesicles.

### Quantitative Analysis of Homologs of Strawberry Allergens

Homologs of so far reported strawberry allergens were identified in the proteomics data by performing a blast search against the *F x ananassa* UniProt database that contains several isoforms of Fra a 1, Fra a 3, and Fra a 4 allergens. The analysis resulted in the detection of the following homologs for each of the three allergens: Fra a 1 (major allergen Pru av 1-like, XP_004296887.1), Fra a 3 (three non-specific lipid transfer proteins, XP_004297800.1, XP_004300702.1, and XP_011466421.1), and Fra a 4 (profilin, XP_004287490.1). Supplementary Table S4 reports the expression levels measured in all samples and their fold change with respect to TPE. While all three known strawberry allergens were expressed in TPE, we could only identify Fra a 1 and Fra a 4 in the vesicle samples by MS-based proteomics analysis. Fold-changes of Fra a 1 and Fra a 4 homologs were measured less than two indicating no significant enrichment for these allergens in the vesicle samples with respect to TPE. Specifically, Fra a 1 was expressed at a lower level in MVs (fold change 0.2) and NVs (fold change 0.5) as in TPE, and the same level in DGUC-NVs. Fra a 4 expression was lower in MVs (fold change 0.4), similar in DGUC-NVs (fold change 0.9), and higher in NVs (fold change 1.5) than in TPE. Thus, quantitative proteomics indicate that while vesicles carry two out of the three strawberry allergens, they were less expressed (Fra a 1) or not significantly enriched (Fra a 4) within the different vesicle populations.

### Identification of IgE-Binding Proteins in Strawberry Vesicle Isolates by the SPHIAa Assay

**FIGURE 3:**
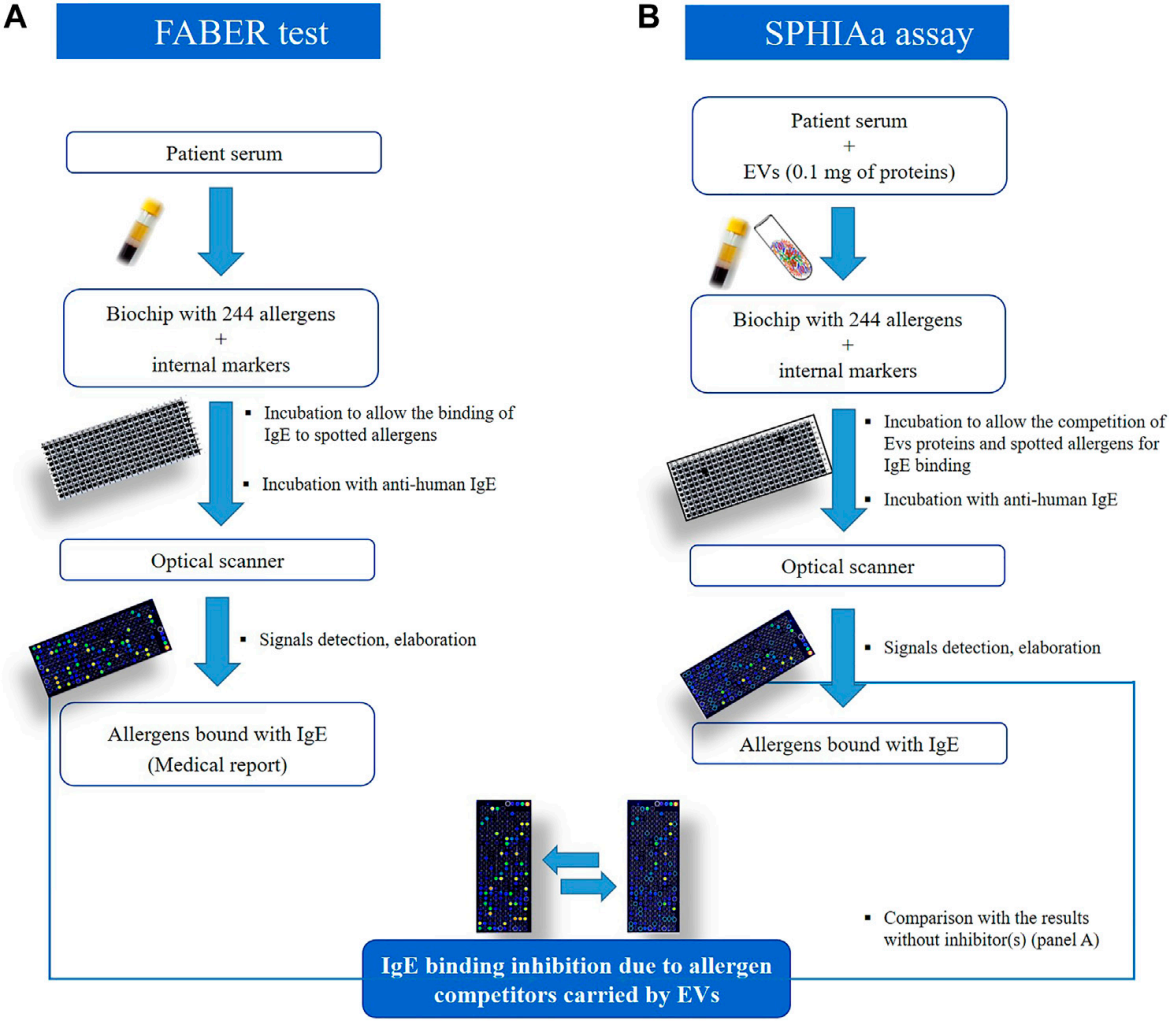
Schematic representation to compare the FABER test on **(A)**, which is used for allergy diagnosis and research purposes, to the procedure used for the Single Point Highest Inhibition Achievable assay (SPHIAa) assay **(B)**.

The presence in strawberry-derived vesicle isolates (MVs, NVs, and DGUC-NVs) of proteins recognized by IgE antibodies contained in the sera of allergic patients was investigated by *in vitro* immunological tests. In particular, the SPHIAa method was used to probe allergens included in the FABER system (Figure 3).

**FIGURE 4:**
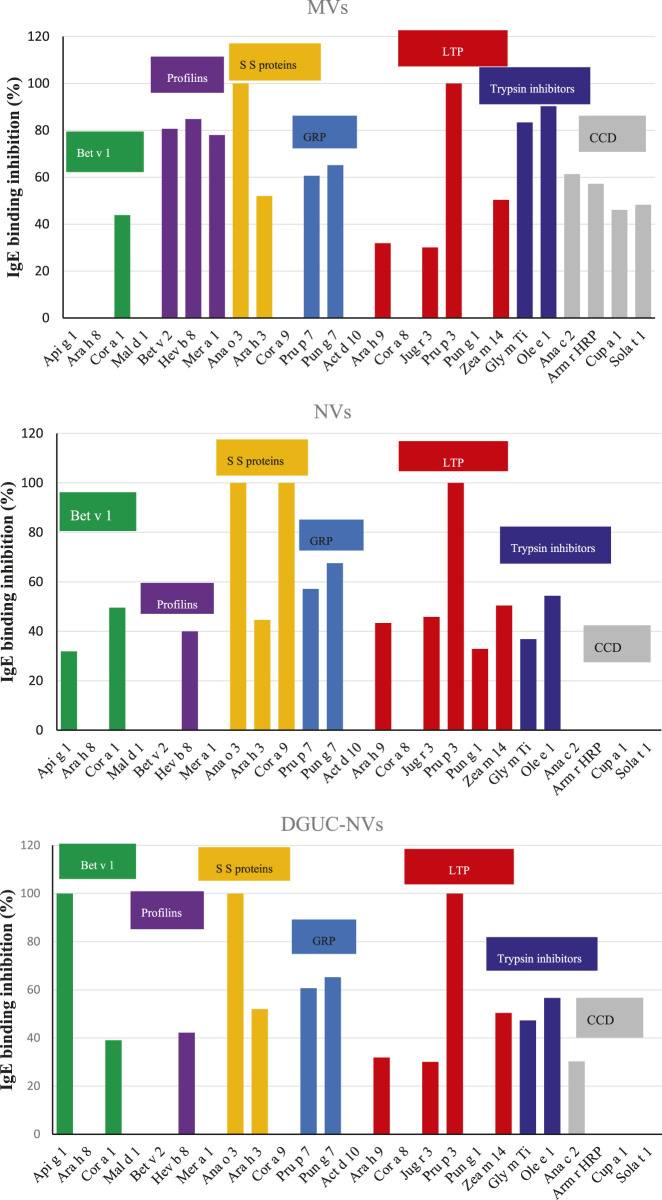
IgE-binding inhibition assays using the FABER testing system with the SPHIAa method and a pool of sera from allergic patients as the provider of specific IgE. The three panels show the percentage of inhibition on the indicated allergens using NVs, MVs, and DGUC-NVs as inhibitors. The allergens belonging to the same protein family have been grouped. The protein families are highlighted with different colors: Bet v 1-like allergens in green, profilins in purple, seed storage proteins in yellow, gibberellin-regulated proteins (GRP) in light blue, LTP in dark red, trypsin inhibitors in blue. In addition, cross-reactive carbohydrate determinant (CCD)-bearing proteins are highlighted in gray.

The isolated strawberry allergens (Fra a 1, Fra a 3, and Fra a 4) were not present on the FABER biochip, but several Bet v1, LTP, and profilin homologs from other sources were available. In the assay, the proteins transported by the vesicles compete with the allergens spotted on the FABER biochip for binding to specific IgE. Vesicle isolates were applied without lysis. Table 2 shows the allergens of relevant allergen families, contained in the FABER biochip, that were analyzed for IgE-binding inhibitions. In particular, we observed inhibition on seven protein allergen groups: Bet v 1-like proteins (Fra a 1), profilins (Fra a 4), seed storage proteins (2S-albumin/11S globulin or legumin-like protein), gibberellin-regulated proteins (GRPs), LTP (Fra a 3), trypsin inhibitors, cross-reactive carbohydrate determinant (CCD) markers and other CCD-bearing allergens. It can be observed that components of the same protein family are inhibited to a different extent (Figure 4) producing different inhibition profiles of MVs, NVs, and DGUC-NVs. Five allergens of the Bet v 1-like family (Bet v 1, Api g 1, Ara h 8, Cor a 1, and Mal d 1) were available in the test system for the SPHIAa assay. The celery Api g 1 was completely inhibited by DGUC-NVs proteins, and it was partially inhibited by NVs, whereas no inhibition at all was produced by the proteins of MVs. This trend is in line with the quantitative data measured by proteomics. The hazelnut Cor a 1 was partially inhibited at a similar extent by all the three vesicles fractions, whereas the peanut, apple, and birch pollen homologous allergens were not inhibited. Among the three analyzed profilins, the latex Hev b 8 is the only one partially inhibited by all the three vesicle fractions, whereas the birch pollen Bet v 2 and Mer a 1 from annual mercury are inhibited at about 80% by MVs only. Three seed storage proteins competed with vesicle proteins for IgE binding. The cashew 2S albumin, Ana o 3, displayed 100% IgE-binding inhibition with all the vesicle fractions, whereas the hazelnut 11S globulin, Cor a 9, was completely inhibited by NVs only. Conversely, the peanut 11S globulin, Ara h 3, was partially inhibited by all the vesicles studied.

**TABLE 2:** Details of plant allergens analyzed for competition in the IgE binding with the proteins contained in the vesicles samples.

Allergen family	Allergen in strawberry	Homologous allergens (in FABER)	Source	Tissue	Specific IgE (FIU)^a^	NCBI accession number(s) of homologs in *F. vesca*	Identification of protein homologs in TPE, MVs, NVs or DGUC-NVs
Bet v 1-like proteins	Fra a 1	Api g 1	Celery	Leaf, Root	4.11	XP_011461765.1	DGUC
		Ara h 8	Peanut	Seed	17.18	XP_004298700.1	DGUC
		Bet v 1	birch	pollen	107.68	XP_004296886.1	TPE
		Cor a 1	Hazelnut	Seed	3.35	XP_004296893.1	MVs and DGUC
		Mal d 1	Apple	Fruit	60.82	XP_004296889.1	MVs and NVs
						XP_004296876.1	TPE and MVs
						XP_004296884.1	All
						XP_004297655.1	All
						XP_004296875.1	All
						XP_004296887.1	All
						XP_004297384.1	All
Profilins	Fra a 4	Bet v 2	Birch	Pollen	37.8	XP_004291152.1	All
		Hev b 8	Rubber tree	Latex	59.16	XP_004287490.1	
		Mer a 1	Annual mercury	Pollen	47.56		
Seed storage proteins (2S albumin/11S globulin	nr	Ana o 3	Cashew	Seed	3.59	XP_004294614.1	All
		Ara h 3	Peanut	Seed	8.69		
		Cor a 9	Hazelnut	Seed	1.84		
Gibberellin-regulated proteins (GRP)	nr	Pru p 7	Peach	Fruit	24.75		
		Pun g 7	Pomegranate	Fruit	19.05		
9K-LTP	Fra a 3	Act d 10	Green kiwifruit	Seed	68.5	XP_004297800.1	TPE
		Ara h 9	Peanut	Seed	20.98	XP_004300702.1	
		Cor a 8	Hazelnut	Seed	86.38	XP_011466421.1	
		Jug r 3	Walnut	Seed	37.26		
		Pru p 3	Peach	Fruit	4.09		
		Pun g 1	Pomegranate	Fruit	61.59		
		Zea m 14	Maize	Seed	25.71		
Trypsin inhibitors	nr	Gly m Ti	Soybean	Seed	14.18		
		Ole e 1	Olive-tree	Pollen	35.82		
CCD markers	nr	Ana c 2	Pineapple	Fruit	91.52	XP_004291075.1	All
		Arm r HRP	Horseradish	Leaf	81.3	XP_004302839.1	
						XP_004294686.1	
Glycosylated allergens	nr	Cup a 1	Cypress	Pollen	86.22		
		Sola t 1	Potato	Tuber	87.47		

Note. The level of IgE (FIU)^a^, specific for the listed allergens and contained in the pool of sera of allergic patients used in the Single Point Highest Inhibition Achievable assay (SPHIAa) experiments with the FABER test, is shown. Components of the same protein family are grouped.

a FIU, FABER International Units, positive value FIU ≥ 0.01; nr, not reported; TPS, total protein extract.

The two analyzed GRP, the peach Pru p 7 and the pomegranate Pun g 7, were partially inhibited by all the three vesicle samples with values around 60%. Six components of the LTP family were available for SPHIa assays. All the vesicle samples produced 100% IgE-binding inhibition on the peach LTP, Pru p 3, and a partial inhibition on the peanut Ara h 9, the walnut Jug r 3, and the maize Zea m 14. The hazelnut Cor a 8 was never inhibited, whereas the pomegranate Pun g 1 showed a value of about 34% with NVs.

The trypsin inhibitors, namely, the soybean Gly m Ti and the olive tree Ole e 1, were inhibited by all three vesicle isolates, but the highest values were produced by MVs. Only MVs caused inhibition on both the CCD markers, the pineapple Ana c 2, and the horseradish Arm r HRP Altmann, 2016. In addition, this isolate caused IgE-binding inhibition on two glycosylated allergens, Cup a 1 and Sola t 1, which are frequently recognized by CCD-specific IgE.

## Identification of Sequence Homologs to known Allergens Enriched in Vesicle Samples

**FIGURE 5:**
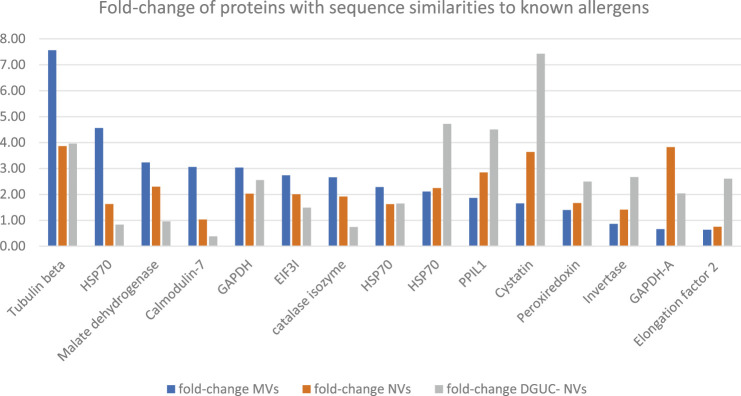
Fold-change of proteins with sequence similarities to known allergens in the COMPARE database and highly expressed in MVs, NVs, or sucrose gradient ultracentrifugation separated NVs fraction. Fold-change with respect to the total protein extract measured by quantitative shotgun proteomics (Supplementary Table S5).

To find homologs to known allergens in strawberry vesicles, a BLAST sequence similarity searching of the quantified proteins (Supplementary Table S2) was performed against the COMPARE database. The analysis identified 56 homologs with 50% or higher sequence identity, and these, together with their expression values, are reported in Supplementary Table S5. Among these proteins, we can find that beta-tubulin (BTUB), glyceraldehyde-3-phosphate dehydrogenase (GAPDH), cysteine proteinase inhibitor (CPI, cystatin), and peptidyl-prolyl cis-trans isomerase 1 (PPIL1) were highly expressed in all vesicle samples (Supplementary Table S5 and Figure 5) compared with TPE. BTUB was reported as an allergen from dust mites, but a recent study reported on plant-specific tubulin as a respiratory allergen from pollen grains too (Ghosh et al., 2015). BTUB was especially highly enriched in the MV isolate (7.6 times higher than in TPE). GAPDH was about threefold enriched in all vesicle samples in comparison with the extract. GAPDH is frequently found to be expressed in EVs. A recent study identified GAPDH as one of the *in vitro* and *in vivo* IgE suppressors present in strawberry extract (Iwamoto et al., 2021). Our study is the first indication that GAPDH could be accumulated in, and perhaps transmitted by, membrane-enclosed vesicles. The sequence homolog of CPI 12 (XP_004307209.1) Act d 4 is a kiwifruit allergen that contributes to the clinical symptoms of kiwifruit allergy (Popovic et al., 2010). The homolog of Act d 4 in strawberry has not yet been reported. However, plant EVs have been reported to carry a complete set of defense molecules; thus, it was not surprising that CPI, an important molecule involved in plant development and defense, was found to accumulate in all strawberry vesicles and especially in the NVs. Bet v 7 from birch pollen and Cat r 1 from periwinkle pollen are considered pan-allergens due to frequently reported cross-reactivity. PPIL1 (XP_004289844.1) of strawberry shows structural similarity with Bet v 7 cyclophilin, and it was enriched in all vesicle samples though at a higher rate in DGUC-NVs.

**FIGURE 6:**
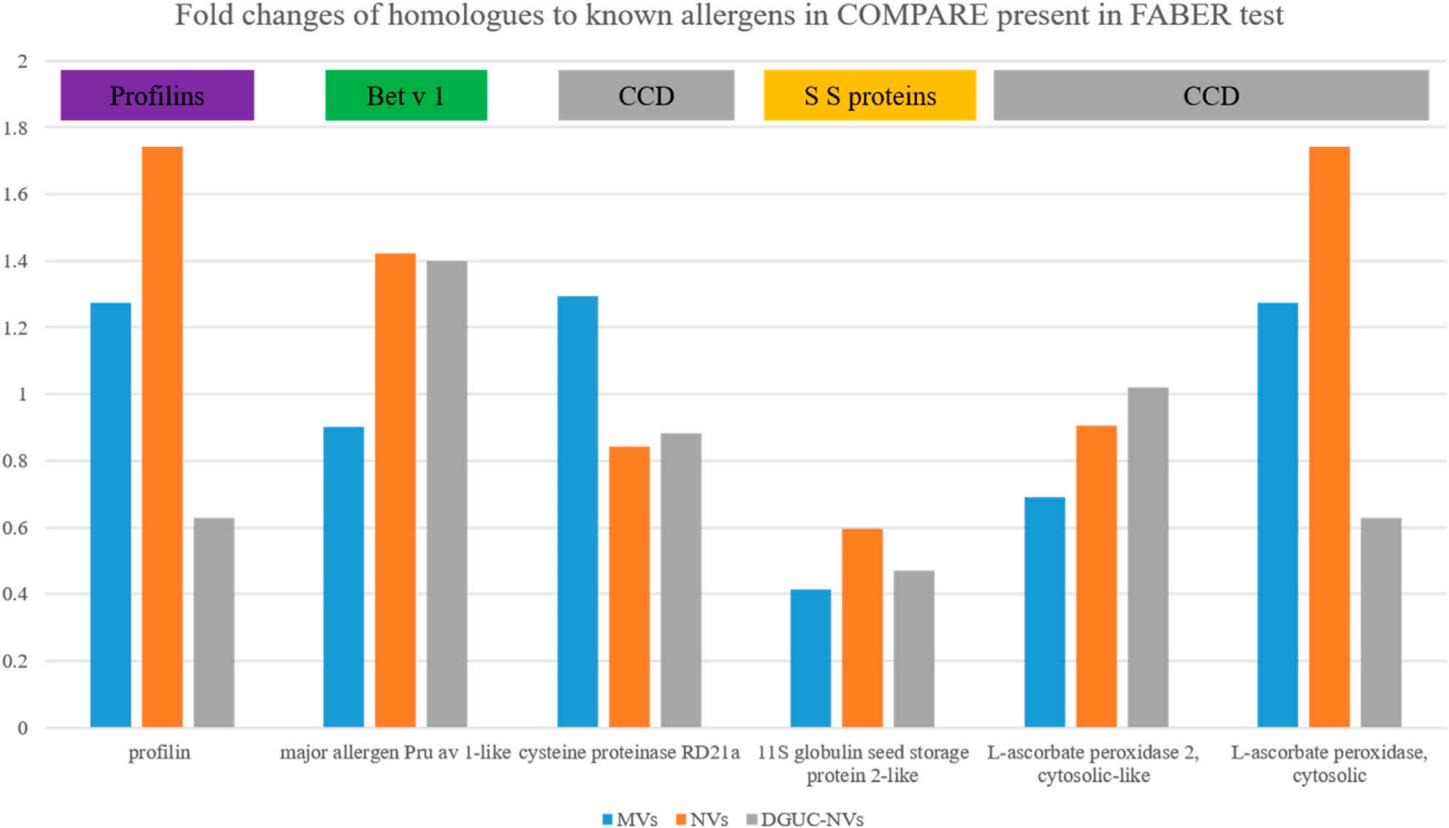
The fold changes measured by quantitative proteomics of the six proteins, homologs to allergens commonly identified in MVs, NVs, and DGUC-NVs (Supplementary Table S5).

To find the sequence homologs to the allergens identified by the SPHIAa *in vitro* immunological tests among the identified proteins (*F. vesca*), we performed a blast search of allergens inhibited in FABER and their isoforms published in the Allergome database against COMPARE database. This resulted in 24 sequences out of which we identified six structurally similar proteins in our proteomics dataset. This comprises the Fra a 4 (profilin), Fra a 1 (Bet v 1), CCD-bearing proteins, and seed storage proteins. Figure 6 shows the fold change of these proteins with respect to TPE measured by label-free quantitative proteomics. While different expression patterns of these proteins were found in the different vesicle populations (MVs, NVs, and DGUC-NVs), it is important to note that their fold changes were less than two in all cases (Figure 6). This indicates that these homologs to allergens measured by FABER are present, but they are not significantly enriched in the vesicle populations.

## Discussion

Here, we isolated three different vesicle populations from the false fruit of strawberries based on size (MVs and NVs) and density (DGUC-NVs). We obtained very high yields that could put a strawberry in an interesting position for the production of nanovesicles from this plant source. NVs were separated by the density gradient to obtain vesicle populations similar to mammalian EVs in density. NTA analysis showed the vesicle character of the samples and proved that MVs, although not considerable, were bigger than NVs. The SDS-PAGE and shotgun proteomic analyses revealed complex protein profiles of all three vesicle isolates studied comparable with that of the total protein extract of strawberries. This is in line with the observations reported in other fruit-derived vesicles, such as tomato (Bokka et al., 2020) and citrus (Pocsfalvi et al., 2018), and share characteristics with mammalian EVs isolated from different sources, such as biofluids and cell cultures. The proteomes of the different vesicle isolates showed overlapping protein contents: 1,234 proteins were commonly expressed (Figure 2B), although in different amounts in all vesicle samples (Supplementary Table S3). However, distinct features could also be recognized since 219, 252, and 111 proteins were exclusively identified in the MVs, NVs, and DGUC-NVs vesicle isolates (Figure 2B). Quantitative proteomics highlighted a set of proteins that were significantly overexpressed in the vesicles isolated from strawberries and which could be useful as markers for their enrichment. Among these, tubulin, HSPs, pyruvate kinase, and ATPases are often reported in both plant-derived vesicles and mammalian EVs.

Proteomics and bioinformatics analysis identified and quantified several proteins in TPE and vesicle isolates with high sequence similarities to known strawberry allergens and allergens from other sources reported in the COMPARE database. Immunological investigations revealed that vesicle isolates from strawberries were carrying proteins bearing functional IgE epitopes. Competition between the proteins of the vesicle isolates and components of several relevant allergen families, included in the FABER biochip, was detected by IgE-binding inhibition experiments. The results obtained showed that the vesicles carry all the three allergens so far described in strawberry, belonging to the protein families of Bet v 1-like protein, (Fra a 1), LTP (Fra a 3), and profilins (Fra a 4). Proteomics identified Fra a 3 in TPE, but not in of the vesicle preparations. This could be explained by its lower than the detectable of this allergen level in the vesicles. Importantly, the quantitative proteomic analysis did not show a significant fold enrichment of the three strawberry allergens in the vesicle samples with respect to TPE. Moreover there, we identified 14 proteins that were homologs of known allergens present in the COMPARE database.

In addition to the already reported allergenic proteins of strawberries, immunological and proteomics results suggest that additional allergenic proteins and homologs to known allergens contained in this food can be carried by vesicles. In particular, IgE-binding proteins, belonging to seed storage proteins, GRP, and trypsin inhibitor allergen families were detected as part of the vesicular cargo.

For instance, all the three vesicle fractions competed with IgE specific for the cashew 2S albumin immobilized on the FABER biochip giving 100% inhibition. In addition, inhibition on peanut and hazelnut 11S globulins was observed.

The immunological investigation also suggests that vesicles of strawberry carry homologous to allergenic GRP. All the vesicle isolates caused a similar partial inhibition (about 60%) IgE-binding inhibition on peach (Pru p 7) and pomegranate (Pun g 7) GRP. The first allergenic protein of the GRP family was discovered quite recently (Tuppo et al., 2013), but later, the number of components reported to cause allergic reactions has increased. Since severe symptoms, including anaphylaxis, have been associated with these proteins, GRP represents a clinically relevant allergen family (Inomata, 2020). The detection of proteins competing with Pru p 7 and Pun g 7 for IgE binding suggests that strawberries could be an additional source of allergenic GRP, and they are part of the protein cargo of the vesicles. Nevertheless, the sensitization revealed by IgE binding is not sufficient to understand the clinical relevance of strawberry GRP, and further studies are required.

In this study, as per our knowledge, the presence of functional allergens was shown for the first time in plant food-derived vesicles. These novel nanomaterials have promising characteristics as native carriers of endogenous substances against inflammation and cancer and they are under exploitation as nanovectors for the delivery of exogenous substances for human health. The allergens detected were not enriched in strawberry vesicles with respect to the fruit extract showing that vesicles are not specific vehicles of these allergens. Before the application of these nanomaterials in human health and nutrition, however, it is important to accumulate information on their possible allergenicity and, thus, carry out a similar analysis in other plant food derived vesicles.

## Data Availability

The datasets presented in this study can be found in online repositories. The names of the repository/repositories and accession number(s) can be found in the article/Supplementary Material.

## References

[B1] Alessandri C. Ferrara R. Bernardi M. L. Zennaro D. Tuppo L. Giangrieco I. (2020). Molecular Approach to a Patient's Tailored Diagnosis of the Oral Allergy Syndrome. Clin. Transl. Allergy 10, 22. 10.1186/s13601-020-00329-8 32551040 PMC7298840

[B2] Alessandri C. Ferrara R. Bernardi M. L. Zennaro D. Tuppo L. Giangrieco I. (2017). Diagnosing Allergic Sensitizations in the Third Millennium: Why Clinicians Should Know Allergen Molecule Structures. Clin. Transl. Allergy 7, 21. 10.1186/s13601-017-0158-7 28725346 PMC5513363

[B3] Altmann F. (2016). Coping with Cross-Reactive Carbohydrate Determinants in Allergy Diagnosis. Allergo J. Int. 25, 98–105. 10.1007/s40629-016-0115-3 27656353 PMC5016538

[B4] Amatori S. Mazzoni L. Alvarez-Suarez J. M. Giampieri F. Gasparrini M. Forbes-Hernandez T. Y. (2016). Polyphenol-rich Strawberry Extract (PRSE) Shows *In Vitro* and *In Vivo* Biological Activity against Invasive Breast Cancer Cells. Sci. Rep. 6, 30917. 10.1038/srep30917 27498973 PMC4976366

[B5] Bokka R. Ramos A. P. Fiume I. Manno M. Raccosta S. Turiák L. (2020). Biomanufacturing of Tomato-Derived Nanovesicles. Foods 9, 1852. 10.3390/foods9121852 PMC7764365 33322632

[B6] Clement E. Lazar I. Attané C. Carrié L. Dauvillier S. Ducoux‐Petit M. (2020). Adipocyte Extracellular Vesicles Carry Enzymes and Fatty Acids that Stimulate Mitochondrial Metabolism and Remodeling in Tumor Cells. EMBO J. 39, e102525. 10.15252/embj.2019102525 31919869 PMC6996584

[B8] Conesa A. Götz S. (2008). Blast2GO: A Comprehensive Suite for Functional Analysis in Plant Genomics. Int. J. Plant Genomics 2008, 1–12. 10.1155/2008/619832 PMC2375974 18483572

[B9] Ghosh N. Sircar G. Saha B. Pandey N. Gupta Bhattacharya S. (2015). Search for Allergens from the Pollen Proteome of Sunflower (Helianthus Annuus L.): A Major Sensitizer for Respiratory Allergy Patients. PLoS One 10, e0138992. 10.1371/journal.pone.0138992 26418046 PMC4587886

[B10] Giangrieco I. Ricciardi T. Alessandri C. Farina L. Crescenzo R. Tuppo L. (2019). ENEA, a Peach and Apricot IgE-Binding Protein Cross-Reacting with the Latex Major Allergen Hev B 5. Mol. Immunol. 112, 347–357. 10.1016/j.molimm.2019.05.007 31254775

[B11] Hyun T. K. Kim J.-S. (2011). Genomic Identification of Putative Allergen Genes in woodland Strawberry (Fragaria Vesca) and Mandarin orange (Citrus Clementina). Plant Omi. J. 4, 428–434. Available at: https://www.pomics.com/kim_hyun_4_7_2011_428_434.pdf .

[B12] Inomata N. (2020). Gibberellin-regulated Protein Allergy: Clinical Features and Cross-Reactivity. Allergol. Int. 69, 11–18. 10.1016/j.alit.2019.10.007 31784246

[B13] Iwamoto A. Inoue Y. Tachibana H. Kawahara H. (2021). Immunomodulatory Effect of Glyceraldehyde-3-Phosphate Dehydrogenase (GAPDH) in Allergic Conditions *In Vitro* and *In Vivo* . Cytotechnology 73, 333–342. 10.1007/s10616-020-00438-z 34149169 PMC8166990

[B14] Karimi N. Cvjetkovic A. Jang S. C. Crescitelli R. Hosseinpour Feizi M. A. Nieuwland R. (2018). Detailed Analysis of the Plasma Extracellular Vesicle Proteome after Separation from Lipoproteins. Cell. Mol. Life Sci. 75, 2873–2886. 10.1007/s00018-018-2773-4 29441425 PMC6021463

[B15] Konoshenko M. Y. Lekchnov E. A. Vlassov A. V. Laktionov P. P. (2018). Isolation of Extracellular Vesicles: General Methodologies and Latest Trends. Biomed. Res. Int. 2018, 1–27. 10.1155/2018/8545347 PMC5831698 29662902

[B16] Mu J. Zhuang X. Wang Q. Jiang H. Deng Z. B. Wang B. (2014). Interspecies Communication between Plant and Mouse Gut Host Cells through Edible Plant Derived Exosome‐like Nanoparticles. Mol. Nutr. Food Res. 58, 1561–1573. 10.1002/mnfr.201300729 24842810 PMC4851829

[B17] Offermann L. R. Schlachter C. R. Perdue M. L. Majorek K. A. He J. Z. Booth W. T. (2016). Structural, Functional, and Immunological Characterization of Profilin Panallergens Amb a 8, Art V 4, and Bet V 2. J. Biol. Chem. 291, 15447–15459. 10.1074/jbc.M116.733659 27231348 PMC4957032

[B18] Pasquariello M. S. Palazzo P. Tuppo L. Liso M. Petriccione M. Rega P. (2012). Analysis of the Potential Allergenicity of Traditional Apple Cultivars by Multiplex Biochip-Based Immunoassay. Food Chem. 135, 219–227. 10.1016/j.foodchem.2012.04.075

[B19] Pathan M. Keerthikumar S. Ang C.-S. Gangoda L. Quek C. Y. J. Williamson N. A. (2015). FunRich: An Open Access Standalone Functional Enrichment and Interaction Network Analysis Tool. Proteomics 15, 2597–2601. 10.1002/pmic.201400515 25921073

[B20] Perut F. Roncuzzi L. Avnet S. Massa A. Zini N. Sabbadini S. (2021). Strawberry-Derived Exosome-like Nanoparticles Prevent Oxidative Stress in Human Mesenchymal Stromal Cells. Biomolecules 11, 87. 10.3390/biom11010087 33445656 PMC7828105

[B21] Pocsfalvi G. Turiák L. Ambrosone A. del Gaudio P. Puska G. Fiume I. (2018). Protein Biocargo of Citrus Fruit-Derived Vesicles Reveals Heterogeneous Transport and Extracellular Vesicle Populations. J. Plant Physiol. 229, 111–121. 10.1016/j.jplph.2018.07.006 30056374

[B22] Popovic M. M. Milovanovic M. Burazer L. Vuckovic O. Hoffmann-Sommergruber K. Knulst A. C. (2010). Cysteine Proteinase Inhibitor Act D 4 Is a Functional Allergen Contributing to the Clinical Symptoms of Kiwifruit Allergy. Mol. Nutr. Food Res. 54, 373–380. 10.1002/mnfr.200900035 19885843

[B23] Stanly C. Fiume I. Capasso G. Pocsfalvi G. (2016). Isolation of Exosome-like Vesicles from Plants by Ultracentrifugation on Sucrose/deuterium Oxide (D2O) Density Cushions. Methods Mol. Biol. 1459, 259–269. 10.1007/978-1-4939-3804-9_18 27665565

[B24] Théry C. Witwer K. W. Aikawa E. Alcaraz M. J. Anderson J. D. Andriantsitohaina R. (2018). Minimal Information for Studies of Extracellular Vesicles 2018 (MISEV2018): a Position Statement of the International Society for Extracellular Vesicles and Update of the MISEV2014 Guidelines. J. Extracell. Vesicles 7, 1. 10.1080/20013078.2018.1535750 PMC6322352 30637094

[B25] Tuppo L. Alessandri C. Giangrieco I. Ciancamerla M. Rafaiani C. Tamburrini M. (2019). Isolation of cypress Gibberellin-Regulated Protein: Analysis of its Structural Features and IgE Binding Competition with Homologous Allergens. Mol. Immunol. 114, 189–195. 10.1016/j.molimm.2019.07.023 31376732

[B26] Tuppo L. Giangrieco I. Alessandri C. Ricciardi T. Rafaiani C. Ciancamerla M. (2018). Pomegranate Chitinase III: Identification of a New Allergen and Analysis of Sensitization Patterns to Chitinases. Mol. Immunol. 103, 89–95. 10.1016/j.molimm.2018.09.009 30241023

[B7] van Ree R. Sapiter Ballerda D. Berin M. C. Beuf L. Chang A. Gadermaier G. (2021). The COMPARE Database: A Public Resource for Allergen Identification, Adapted for Continuous Improvement. Front. Allergy 2, 39. 10.3389/falgy.2021.700533 PMC8974746 35386979

[B27] Yakhlef M. Giangrieco I. Ciardiello M. A. Fiume I. Mari A. Souiki L. (2021). Potential Allergenicity of Medicago Sativa Investigated by a Combined IgE ‐binding Inhibition, Proteomics and In Silico Approach. J. Sci. Food Agric. 101, 1182–1192. 10.1002/jsfa.10730 32790067

[B28] Zhang J. Wang X. Yu O. Tang J. Gu X. Wan X. (2011). Metabolic Profiling of Strawberry (Fragaria×ananassa Duch.) during Fruit Development and Maturation. J. Exp. Bot. 62, 1103–1118. 10.1093/jxb/erq343 21041374

