# Supplementary figures and images for: Crosstalk Between the Immune System and Plant-Derived Nanovesicles: A Study of Allergen Transporting
Source: Front Bioeng Biotechnol. 2021 Nov 26;9:760730. doi: 10.3389/fbioe.2021.760730 (PMC8662998; doi:10.3389/fbioe.2021.760730)

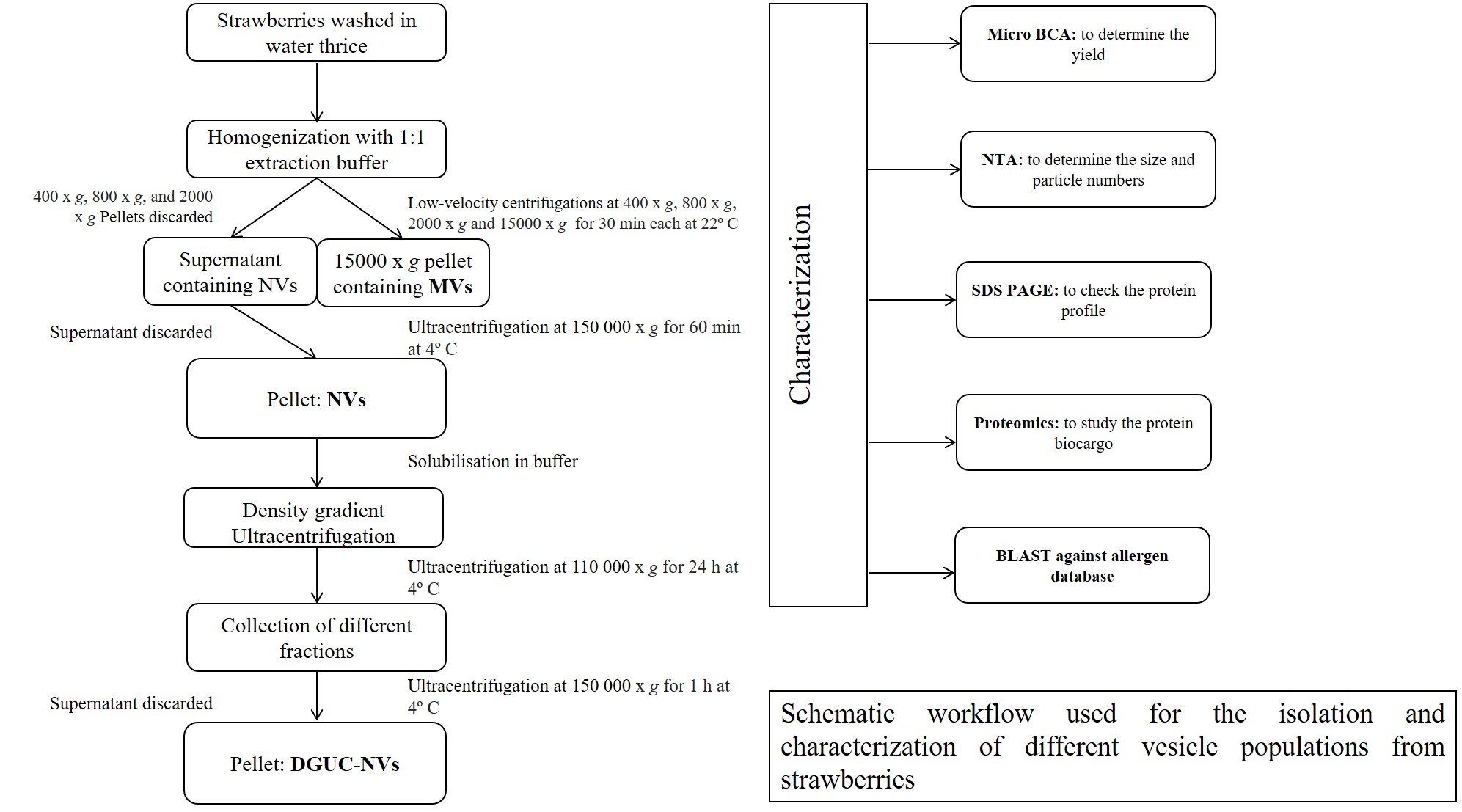

Supplement: Supplementary file 3 [file Image1.JPEG]
